# Children and young people’s concerns and needs relating to their use of health technology to self-manage long-term conditions: a scoping review

**DOI:** 10.1136/archdischild-2020-319103

**Published:** 2020-05-22

**Authors:** Sarah Blower, Veronica Swallow, Camila Maturana, Simon Stones, Robert Phillips, Paul Dimitri, Zoe Marshman, Peter Knapp, Alexandra Dean, Steven Higgins, Ian Kellar, Penny Curtis, Nathaniel Mills, Jacqueline Martin-Kerry

**Affiliations:** 1 Department of Health Sciences, University of York, York, UK; 2 College of Health, Wellbeing & Life Sciences, Sheffield Hallam University, Sheffield, South Yorkshire, UK; 3 York Trials Unit, University of York, York, North Yorkshire, UK; 4 School of Healthcare, University of Leeds, Leeds, West Yorkshire, UK; 5 Centre for Reviews and Dissemination, University of York, York, North Yorkshire, UK; 6 NIHR Children and Young People MedTech Cooperative, Sheffield Children's Hospital NHS Foundation Trust, Sheffield, Sheffield, UK; 7 School of Clinical Dentistry, The University of Sheffield, Sheffield, Sheffield, UK; 8 Department of Health Sciences and Hull York Medical School, University of York, York, North Yorkshire, UK; 9 School of Education, University of Durham, Durham, UK; 10 School of Psychology, University of Leeds, Leeds, West Yorkshire, UK; 11 School of Nursing and Midwifery, The University of Sheffield, Sheffield, Sheffield, UK; 12 NIHR Children and Young People MedTech Co-operative and NIHR Devices for Dignity MedTech Co-operative, Sheffield Children's NHS Trust, Sheffield, UK

**Keywords:** technology, adolescent health

## Abstract

**Background:**

The use of patient-facing health technologies to manage long-term conditions is increasing; however, children and young people may have particular concerns or needs before deciding to use different health technologies.

**Aims:**

To identify children and young people’s reported concerns or needs in relation to using health technologies to self-manage long-term conditions.

**Methods:**

A scoping review was conducted. We searched MEDLINE, PsycINFO and CINAHL in February 2019. Searches were limited to papers published between January 2008 and February 2019. We included any health technology used to manage long-term conditions. A thematic synthesis of the data from the included studies was undertaken. We engaged children with long-term conditions (and parents) to support review design, interpretation of findings and development of recommendations.

**Results:**

Thirty-eight journal articles were included, describing concerns or needs expressed by n=970 children and/or young people aged 5–18 years. Most included studies were undertaken in high-income countries with children aged 11 years and older. Studies examined concerns with mobile applications (n=14), internet (n=9), social media (n=3), interactive online treatment programmes (n=3), telehealth (n=1), devices (n=3) or a combination (n=5). Children and young people’s main concerns were labelling and identity; accessibility; privacy and reliability; and trustworthiness of information.

**Discussion:**

This review highlights important concerns that children and young people may have before using technology to self-manage their long-term condition. In future, research should involve children and young people throughout the development of technology, from identifying their unmet needs through to design and evaluation of interventions.

**What is already known on this topic?**

- The use of patient-facing technologies for children and young people (CYP) to self-manage long-term conditions (LTCs) is rapidly increasing.
- There are many studies exploring the use or development of new health technology but few that explored CYP’s concerns about the use of this technology.
- It is important to obtain stakeholders’ views (particularly CYP’s) about their use of technologies or treatments.

**What this study adds?**

- We have identified key concerns of CYP about their use of health technology to self-manage LTCs.
- Concerns included labelling and identity; accessibility; privacy and reliability; and trustworthiness.
- It is important to understand and address these concerns as they are potential barriers to engagement with health technologies

## Background

Patient-facing health technologies (eg, virtual reality, augmented reality, telehealth and medical devices) have the potential to address key healthcare challenges, and their use is rapidly expanding.^1^ Increasingly, adults with long-term conditions (LTCs) self-manage their health,^2^ sometimes with remote clinical support and monitoring. This approach could reduce health system burden, while offering convenience for clinician–patient engagement.^3^ There is growing interest in the use of technologies to support children and young people (CYP) with LTCs.^4^

Involving CYP with LTCs in developing and using health technologies provides opportunities for enhancing their health and well-being.^1^ To date, there is limited research into the challenges of using technology and concerns felt by end-users, particularly CYP. Recent systematic reviews highlight privacy and security issues associated with the use of mobile health applications (apps) for CYP^4–6^ and CYP wanting access to safe, moderated forums to communicate with peers.^7^ For example, the Brushing RemInder 4 Good Oral HealTh (BRIGHT) trial^8^ used a short messaging service to encourage CYP to brush their teeth. During the intervention development and trial design, CYP expressed concerns over who could access their mobile phone numbers and how they could stop receiving text messages. Recent studies^7 9^ suggest that CYP may require specific information and guidance on privacy, security and data confidentiality before participating in research involving healthcare technologies. This scoping review and associated stakeholder consultation aimed to identify empirical research reporting CYP’s concerns and needs relating to the use of health technologies to self-manage LTCs and develop recommendations for technology developers and researchers.

## Methods

A scoping review^10^ was undertaken without quality assessments.^11^

### Search strategy

Ovid MEDLINE, PsycINFO and CINAHL were searched in February 2019 using a strategy developed with an information specialist and modified for each database (see online supplementary appendix 1). The search was limited to papers published between January 2008 and February 2019 to ensure relevance to current health technologies.

10.1136/archdischild-2020-319103.supp1 Supplementary data

### Eligibility

table 1 outlines the review inclusion and exclusion criteria.

**Table 1:** Eligibility criteria for studies within this review

Inclusion criteria	Exclusion criteria
**Population:** CYP with physical and/or mental LTCs aged up to and including 18 years (no lower age limit). LTCs were defined as ‘those conditions for which there is currently no cure, and which are managed with drugs and other treatment’.^78^ **Concept:** concerns and needs of CYP in relation to health technology including privacy, stigma, security, views about barriers to how they use health technology and any information that CYP suggested they needed to know before using health technology. **Context:** the focus was on health technologies that CYP engage or interact with to manage LTCs. Health technologies included: mobile apps; virtual and augmented reality; telehealth; digital health; digitised medical devices; gamification/health gaming; receiving health information via SMS (digital health education messages); patient care/monitoring wearables; remote monitoring; consumer products (eg, Fitbit); and social media. All settings (eg, home, hospital and clinic) and countries were included. Studies examining hypothetical (prospective) use, (how CYP *may* use the technology and what their concerns *may* be) and those studying retrospectively (after CYP *had* used the technology, either in real life situation or in a user-testing scenario), were included. **Study design:** qualitative, surveys/questionnaires, feasibility, acceptability, user-testing/usability and mixed methods (including any of these study designs undertaken within trials), where data from those <18 years or younger could be extracted.	Studies were excluded if they: (1) did not involve CYP with LTCs; (2) only explored parents’ or clinicians’ views, experiences, use or concerns about a health technology; (3) explored use of health technologies to manage acute conditions, diagnosis or for one-off measurements; (4) included technologies to enhance mobility, senses or provide medications (eg, hearing aids, mobility aids and prostheses); (5) exclusively included CYP aged over 18 years; (6) did not separate CYP’s and adults’ data within the study; and (7) were not published in English.

CYP, children and young people; LTCs, long-term conditions; SMS, short messaging service.

### Study selection

Records were deduplicated in Endnote and managed using Covidence. JM-K screened title and abstracts, with 20% of records double-screened (SB and VS). Agreement rate and Cohen kappa coefficients were calculated to measure inter-rater reliability. Three reviewers (JM-K, SB and CM) undertook screening of full-text records independently. When uncertainty about inclusion arose, articles were discussed (JM-K, CM, SB and AD) until a consensus was reached.

### Data extraction

Data were extracted by JM-K (with AD and CM each independently replicating extraction of 50% of the studies) using a prepiloted template. Data extracted included: lead author; year of publication; country; study participant details (age, number, sex and LTC); study design; setting where technology was used; retrospective or prospective use; concerns or information needed before using the technology; whether CYP were involved in the scoping or design of the technology; and any quotations to support the concerns extracted.

### Data synthesis

Bubble plots highlight patterns and gaps in data and identify the number of included studies by country and publication year. Thematic analysis of the findings of each study was undertaken. JM-K and SB read through extracted qualitative (quotations and interpretation from the primary study authors) and quantitative data to identify concerns and needs and assign themes.^12^

### Stakeholder consultation

Throughout the project, we engaged with CYP and parent stakeholders who had used health technologies to manage LTCs. To explore the context for this review from the perspective of CYP (April 2019), JM-K and SS facilitated a discussion with (n=4) stakeholders, two CYP aged 13 and 15 years and their mothers, to determine their views on concerns and informational needs.

Following the review (October 2019), we shared the findings with CYP and parents from the NIHR Generation R Young Persons’ Advisory Group (YPAG). The consultation was a face-to-face meeting with 15 CYP (age 9–18 years) and 4 parents (who have children with LTC). Participants noted and discussed findings that interested or surprised them. Participants were invited to make recommendations for health professionals developing self-management support health technologies (based on the review findings) on Post-it notes and discuss these within the group. The outcomes of this discussion supplemented the review findings and informed the recommendations.

## Results

### Study selection

**Figure 1:**
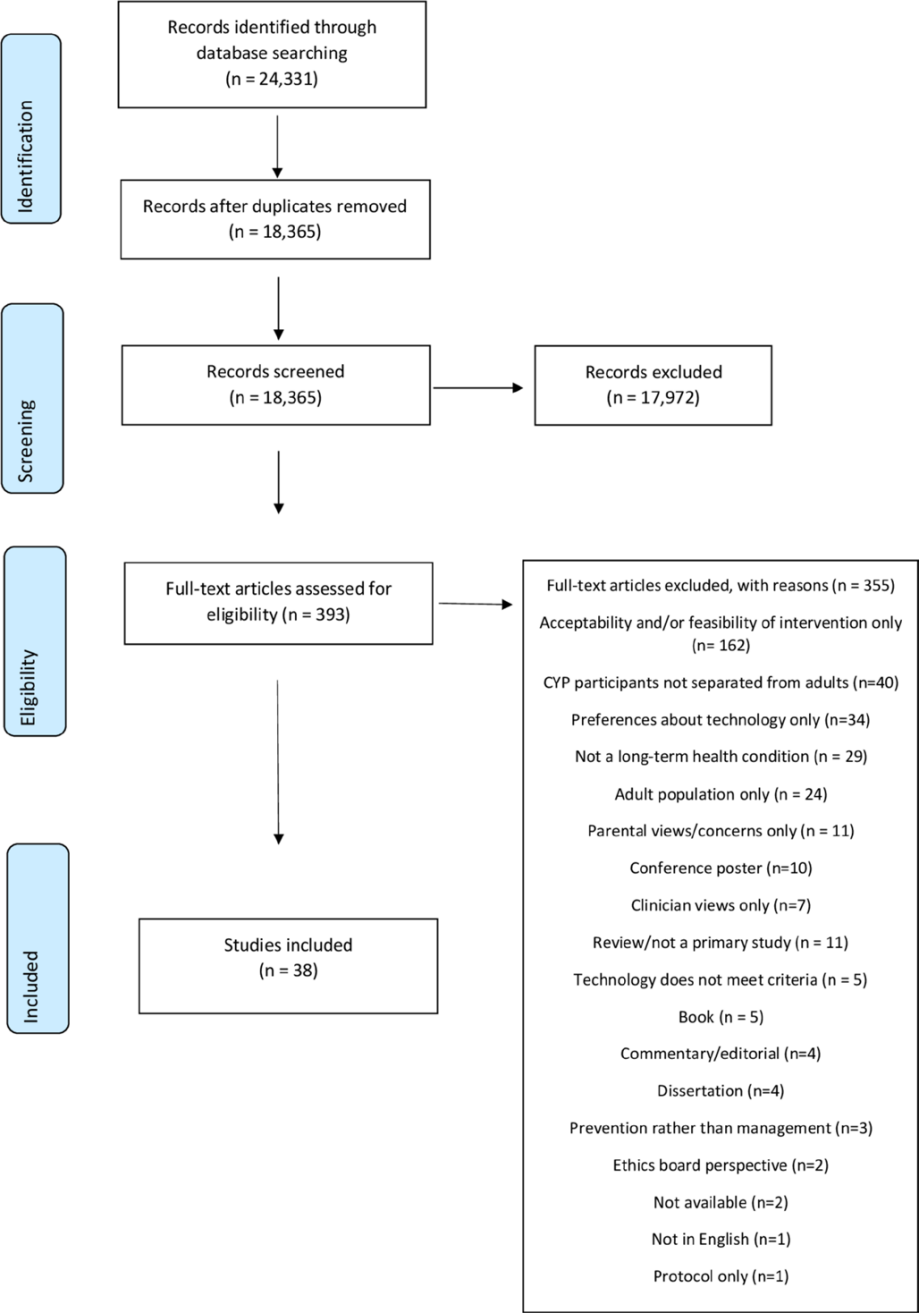
PRISMA flow chart. CYP, children and young people; PRISMA, Preferred Reporting Items for Systematic Reviews and Meta-Analyses.

A total of 18 365 unique records were identified through the electronic searches. There was a 95% agreement rate in the 3673 double-screened abstracts(moderate kappa agreements). No potentially eligible studies were missed. Single screening was undertaken for the remaining 14 692 records. Many excluded papers did not include CYP’s concerns or perspectives (eg, only proxy views from parents or clinicians), or reported the technology use outside the scope of this review. Thirty-eight studies were included (see figure 1).

### Characteristics of included studies

**Figure 2:**
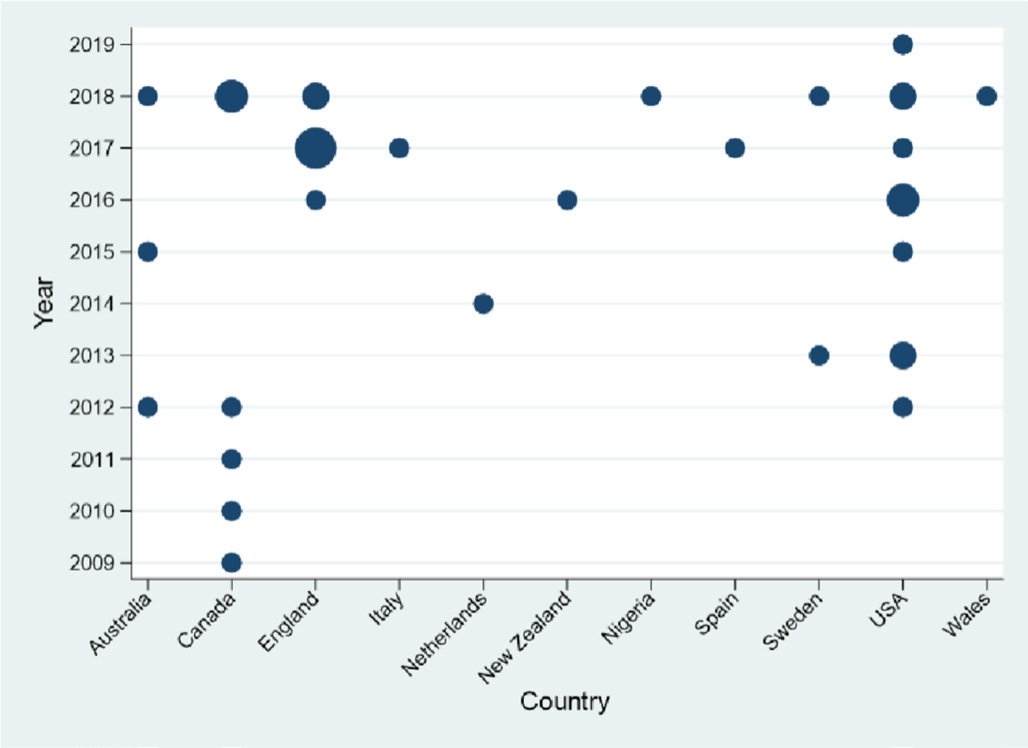
Included studies by publication date and country.

All studies (table 2) were published between 2009 and 2019 and undertaken in Australia^13–15^ (n=3), Canada^16–22^ (n=7), England^23–31^ (n=9), Italy^32^ (n=1), the Netherlands^33^ (n=1), New Zealand^34^ (n=1), Nigeria^35^ (n=1), Spain^36^ (n=1), Sweden^37 38^ (n=2), USA^39–49^ (n=11) and Wales^50^ (n=1). Studies included CYP with the following LTCs: asthma (n=7), type 1 diabetes (n=5), chronic kidney disease (n=3), cancer (n=3), obesity (n=3), cerebral palsy/spina bifida (n=2), attention deficit hyperactivity disorder (ADHD) (n=2) and HIV, idiopathic scoliosis, colorectal conditions, chronic fatigue syndrome/myalgic encephalitis with n=1 study each. Figure 2 shows the distribution of studies by country and publication date.

**Table 2:** Summary of included studies (n=38)

Lead author and year study published	Study design	Country of study	Mean age (years)	Study participants within age range (total sample size)	Study participants' female (%)	Study participants: LTC	CYP involved in the design of the technology?
Barnfather (2011)	Qualitative (individual interviews)	Canada	14.6	22* (27)	12 (44.4)*	Cerebral palsy and spina bifida.	Yes
Bevan Jones (2018)	Qualitative (interviews and focus groups)	Wales	15.85†	11 (33)	7 (64)	Depression.	Yes
Boydell (2010)	Qualitative (individual interviews)	Canada	NR	30 (30)	13 (43.3)	Variety of mental health conditions and neurodevelopmental disorders.	No
Bradford (2015)	Qualitative (focus group discussions)	Australia	NR	17 (129)	9 (53)	Mental health.	No
Brigden (2018)	Qualitative (individual interviews)	England	14.89	9	6 (66.6)	Chronic fatigue syndrome and myalgic encephalomyelitis.	Yes
Britto (2012)	Pilot or feasibility study (questionnaires)	USA	15.2	12‡ (19)	10 (52.6)	Asthma.	No
Cafazzo (2012)	Codesign plus clinical pilot of intervention (interviews and questionnaires)	Canada	14.9	6 involved in design (26 in total within full study)	NR	Type 1 diabetes.	Yes
Cai (2017)	Qualitative (interviews and focus groups)	England	NR	29	19 (65.5)	Juvenile idiopathic arthritis.	Yes
Carpenter (2016)	Qualitative (individual interviews)	USA	14.7	20	9 (45)	Asthma.	No
Clark (2018)	Qualitative (interviews)	Australia	15.2	8 (29)	0 (0)	Anxiety (with or without depression).	No
Dominguez (2017)	Qualitative (interviews) plus questionnaire	Spain	18.7	9 (20)	8 (88.9)	Cancer.	No
Donzelli (2017)	Survey/questionnaire	Italy	14.65	336 (364)	301 (82.7)§	Idiopathic scoliosis.	Yes
Dulli (2018)	Pilot or feasibility study (qualitative and questionnaire)	Nigeria	NR	41	22 (53) – total	HIV.	No
Holmberg (2018)	Qualitative (individual interviews)	Sweden	NR	20	11 (55)	Obesity.	No
Howard (2017)	Usability/user testing (questionnaires and interviews)	England	13.4	7	2 (28.6)	Asthma.	Yes
Huby (2017)	Qualitative (individual interviews)	England	NR	26	12 (46.2)	Chronic kidney disease.	Yes
Jibb (2018)	Pilot study (including interviews and questionnaires)	Canada	NR	20 in qual (40 in larger study)	9 (45)	Cancer.	Yes
Knibbe (2018)	Qualitative (focus group discussions)	Canada	14.4†	8	5 (62.5)	Cerebral palsy.	No
Maurice-Stam (2014)	Pilot study (including questionnaires)	The Netherlands	NR	12 (12)	NR	Cancer.	No
Mulvaney (2013)	Survey/questionnaire	USA	15.2	53	31 (58)	Asthma.	No
Nicholas (2009)	Qualitative (individual interviews)	Canada	15	10 (24)	NR	Chronic kidney disease.	Yes
Nightingale (2017)	Qualitative (individual and joint interviews)	England	NR	17	8 (47.1)	Chronic kidney disease.	Yes
Nordfeldt (2013)	Qualitative (focus group discussions)	Sweden	NR	24 (24)	11 (45.8)	Type 1 diabetes.	No
Powell (2017)	Qualitative (interviews)	England	9.6†	5 (5)	2 (40)	ADHD.	No
Ramsey (2018)	Qualitative (individual interviews)	USA	15.4	20	10 (50)	Asthma.	No
Raval (2017)	Qualitative (joint interviews)	USA	NR	2 (6)	NR	Colorectal diseases.	No
Rivera (2018)	Qualitative (focus groups) plus questionnaires	Canada	14.7	19	13 (68)	Obesity.	Yes
Roberts (2016)	Qualitative (individual and joint interviews) plus questionnaire	USA	14.7	20	9 (45)	Asthma.	No
Schneider (2019)	Usability/user testing (including qualitative)	USA	14.4	20 (20)	11 (55)	Asthma.	Yes
Simons (2016)	Qualitative (focus group discussions) plus questionnaires	England	NR	8 (8)	1 (12.5)	ADHD.	Yes
Stewart (2018)	Qualitative (individual interviews)	England	12.86	8	5 (62.5)	Asthma.	No
Thabrew (2016)	Qualitative (focus group discussions)	New Zealand	12.55†	22	10¶ (45.5)	Variety of long-term physical conditions.	No
Vaala (2018)	Survey/questionnaire	USA	NR	134	75 (56)	Type 1 diabetes.	No
Van Rensburg (2016)	Qualitative (individual interviews)	USA	16.1†	20 (20)	15 (75)	Variety of mental health conditions and neurodevelopmental disorders.	No
Waite-Jones (2018)	Qualitative (interviews and focus groups)	England	13.6†	9	9 (81.8)	Juvenile arthritis.	Yes
Woolford (2013)	Qualitative (interviews and focus groups)	USA	16	11	8 (73)**	Obesity.	No
Wuthrich (2012)	RCT (including questionnaire)	Australia	14.6	24 (43)	16 (66.7)	Anxiety.	Yes
Yi-Frazier (2015)	Qualitative (interviews and focus groups)	USA	16.4	20 (20)††	13 (65)	Type 1 diabetes.	No

*27 in total signed up and 22 participated in the qualitative research; subsequent percentages are % of total enrolled.

†Mean age not reported in the original study but calculated from raw data.

‡Only intervention participants (these are the only participants who provided concerns).

§These figures relate to the 364 approached not the 336 who participated in the study.

¶Estimate calculated from proportions provided in the study.

**Percentages reported in the study appear incorrect so have been adjusted in this table.

††20 CYP were enrolled but only 10 had individual interviews and 5 attended a focus group.

ADHD, attention deficit hyperactivity disorder; CYP, children and young people; LTC, long-term condition; RCT, randomised controlled trial.

Most studies were exclusively qualitative (n=26, 68%),^13 14 16 17 20–24 26 27 29–31 34 36–38 40 42–44 47–50^ while other study designs such as user testing, pilot or feasibility studies and one randomised controlled trial each included some qualitative data (n=12, 32%).^15 18 19 25 32 33 35 39 41 45 46^ Only seven studies included participants under 11 years.^17 24 26–28 31 34^ The age range of CYP represented was 5–18 years.

Technologies were categorised using a previously reported typology^51^: internet (eg, websites, forums, chat rooms and e-tools) (n=9)^13 16 23 26 33–35 37 46^; social media (dedicated platforms, eg, YouTube, Twitter, Facebook and Instagram) (n=3)^47–49^; mHealth (eg, mobile phone apps and text messaging) (n=14)^18 19 22 24 28 29 31 39–45^; telehealth (eg, video conferencing and telephone consultations) (n=1)^17^; interactive online treatment programmes (n=3)^14 15 50^; and devices (eg, wearables and other devices/hardware)^25 30 32^ (n=3). Five studies involved combinations of technologies.^20 21 27 36 38^

### Concerns and needs expressed by CYP

Regardless of technology type, many concerns reported by CYP were similar across studies (see table 3). There were four overarching themes, summarised below, with quotations illustrating key concerns in the words of CYP themselves (table 4). Full list of quotations per study is provided in online supplementary appendix 2.

10.1136/archdischild-2020-319103.supp2 Supplementary data

**Table 3:** Summary of technologies and related concerns raised by CYP

Lead author and date	Age range (years)	Study participants: long-term health condition	Type of technology and brief description	Setting (where technology was studied)	Use of technology	Concerns
Barnfather (2011)	12–18	Cerebral palsy and spina bifida.	Internet (online support).	Home for 25 sessions.	Retrospective.	Stigma/grouping by condition. Noise within chat room. Usability (age appropriateness – too broad an age range).
Bevan-Jones (2018)	13–18	Depression.	Interactive online treatment programme (psychoeducation multimedia programme: MoodHwb).	Discussed during interviews and focus groups.	Prospective.	Security. Confidentiality. Discomfort/unease with technology.
Boydell (2010)	7–18	Variety of mental health conditions and neurodevelopmental disorders.	Telehealth (telepsychiatry).	Clinic (interviewed after teleconsultation).	Retrospective.	Discomfort/unease with technology. Privacy – not wanting others to see or know. Difficulty forming therapeutic relationship due to format (time, not knowing the person).
Bradford (2015)	12–18*	Mental health.	Internet (electronic mental health assessment).	Hypothetical (e-tool described in interviews).	Prospective.	Privacy – not wanting others to see or know. Data security. Fear of misinterpretation. Permanence of written information. Discomfort/unease with technology.
Brigden (2018)	12–17	Chronic fatigue syndrome and myalgic encephalomyelitis.	Internet (online resources).	Discussion of past use of online resources during interviews.	Retrospective.	Trustworthiness of information – needs to be ‘official’ or ‘reliable’. Usability of technology (age appropriate; no jargon).
Britto (2012)	13–18	Asthma.	mHealth (text messaging on mobile phone).	Daily life (home, school and so on) for 3 months.	Retrospective.	Privacy – not wanting others to see or know. Data security. Information being misinterpreted. Permanence of written information. Discomfort/unease with technology.
Cafazzo (2012)	12–16	Type 1 diabetes.	mHealth (smartphone app).	Daily life (home, school and so on).	Retrospective.	Stigma. Privacy – not wanting others to see or know. Functionality of technology.
Cai (2017)	10–18*	Juvenile idiopathic arthritis.	mHealth (smartphone app).	Clinic.	Retrospective.	Privacy – not wanting others to see or know. Data security. Control over how their data are shared.
Carpenter (2016)	12–16	Asthma.	mHealth (smartphone apps).		Retrospective.	Privacy (not wanting others to see or know).
Clark (2018)	12–18	Anxiety (with or without depression).	Interactive online treatment programme (online anxiety disorder treatment programme).	Psychology clinics, school or participant’s house.	Prospective.	Stigma of condition and identification through technology use. Confidentiality. Control over decisions made.
Dominguez (2017)	14–18*	Cancer.	Internet and social media (internet searches about LTC; Facebook, Twitter and Instagram; also blogs).	Interviews – discussion about technology.	Prospective.	Information being negative or unreliable. Usability of technology (age-appropriate language; no jargon). Discomfort/unease with technology.
Donzelli (2017)	NR	Idiopathic scoliosis.	Device (thermobrace plus sensor with reading software).	Daily life (survey in waiting room).	Retrospective.	Control over how their data are shared.
Dulli (2018)	15–18*	HIV.	Internet (online support group).	Daily life (home, school and so on).	Retrospective.	Stigma. Privacy – not wanting others to see or know.
Holmberg (2018)	13–16	Obesity.	Internet (online weight, food and health information).	Discussion about past use in interviews.	Retrospective.	Trustworthiness of information. Realistic information and images need to be used.
Howard (2017)	11–16	Asthma.	Device (electronic monitoring device).	Home.	Retrospective.	Control over how their data are shared. Stigma. Privacy – not wanting others to see or know.
Huby (2017)	5–17	Chronic kidney disease (CKD).	Internet (web-based support for CKD).	Interviews conducted in hospital.	Prospective.	Access to technology (Wi-Fi). Age-appropriateness needed for technology. Trustworthiness of information. Privacy – not wanting others to see or know. Data security.
Jibb (2018)	12–17	Cancer.	mHealth (smartphone app).	Home use for 28 days.	Retrospective.	Responsiveness of healthcare professionals.
Knibbe (2018)	12–18	Cerebral palsy.	Internet, social media, mHealth (Facebook, Youtube, pedometer, fitness app and active video games).	Hospital.	Prospective.	Inclusivity of people with conditions. Stigma (cyberbullying). Privacy – not wanting others to see or know.
Maurice-Stam (2014)	11–17	Cancer.	Internet (website with secure chat room).	Not specified but outside of clinic.	Retrospective.	Privacy – not wanting others to see or know.
Mulvaney (2013)	12–18	Asthma.	mHealth (using phone to monitor asthma).	Daily life (home, school and so on).	Retrospective.	Access to technology (within school).
Nicholas (2009)	NR	Chronic kidney disease.	Internet (email and online social support network).	Daily life (home, school and so on).	Retrospective.	Privacy – not wanting others to see or know. Control over how their data is shared. Unease/discomfort with technology.
Nightingale (2017)	5–18	Chronic kidney disease.	Internet and mHealth (apps and websites).	During interviews.	Prospective.	Trustworthiness of information/technology. Access to technology (finding information). Functionality of technology – data on phone. Age appropriateness (gamification). Unease/discomfort with technology.
Nordfeldt (2013)	10–17	Type 1 diabetes.	Internet and social media (broad definition).	Clinic (focus groups).	Prospective.	Trustworthiness of information/technology. Control over who they share their data with. Usability of technology (age-appropriate language). Privacy – others seeing or knowing. Discomfort/unease with technology.
Powell (2017)	8–13	ADHD.	mHealth (smartphone apps).	Interview location (participant's home).	Retrospective.	Functionality of technology. Usability of technology (age appropriate). Access to technology (cost).
Ramsey (2018)	13–18	Asthma.	mHealth (smartphone apps).	Some use in real life; some hypothetical use in interviews.	Prospective.	Control over how their data is shared.
Raval (2017)	? NR (3-16)	Colorectal diseases.	mHealth (smartphone apps).	During interviews (discussion about apps).	Prospective.	Stigma. Privacy – not wanting others to see or know. Usability of technology. Condition-specific technology. Functionality of technology (data on phone).
Rivera (2018)	12–18	Obesity.	mHealth (smartphone apps).	Discussion about apps in focus groups.	Prospective.	Burden of tracking details. Privacy – not wanting others to see or know.
Roberts (2016)	12–16	Asthma.	mHealth (smartphone apps).	Daily life (home, school and so on).	Retrospective.	Privacy – not wanting others to see or know. Stigma/grouping by condition.
Schneider (2019)	12–17	Asthma.	mHealth (smartphone apps).	Daily life (home, school and so on).	Retrospective.	Functionality of technology. Access to technology (school and data).
Simons (2016)	12–13	ADHD.	mHealth (text message and app for remote monitoring).	During focus groups - discussion about technology.	Prospective.	Access to technology (school and WiFi). Trustworthiness of information/technology.
Stewart (2018)	11–15	Asthma.	Device (electronic monitoring devices).	Daily life (home, school and so on).	Retrospective.	Privacy – not wanting others to see or know. Being monitored or watched. Fear of misinterpretation.
Thabrew (2016)	8–17	Variety of physical conditions.	Internet and interactive online treatment programmes (online support and e-therapy).	Discussed in focus groups (hospital).	Prospective.	Usability of technology (age-appropriateness).
Vaala (2018)	13–17	Type 1 diabetes.	Internet (online questionnaire to sharing personal data with peers).	Clinic.	Prospective.	Control over how their data is shared.
van Rensburg (2016)	14–18	Variety of mental health conditions and neurodevelopmental disorders.	Social media (broad but did specifically include facebook).	n/a	Prospective.	Responsiveness of healthcare professionals. Fear of misinterpretation. Being monitored or watched.
Waite-Jones (2018)	10–18	Juvenile arthritis.	mHealth (smartphone apps).	Discussion in focus groups in clinic.	Prospective.	Security. Control over who how their data is shared. Access to technology (cost). Usability (age-appropriateness).
Woolford (2013)	13–18	Obesity.	Social media (Facebook).	Discussion in focus groups.	Prospective.	Privacy – not wanting others to see or know. Negative content. Stigma.
Wuthrich (2012)	14–17	Anxiety.	Interactive online treatment programme (Cool Teens cCBT).	Daily life (home, school and so on).	Retrospective.	Privacy – sharing personal data.
Yi-Frazier (2015)	14–18	Type 1 diabetes.	Social media (Instagram).	Daily life (home, school and so on).	Retrospective and prospective.	Privacy – not wanting others to see or know. Access to technology (smartphone).

*Age range of total sample exceeded 18 years, but reviewers were able to isolate data pertaining only to CYP whose age range met our inclusion criteria.

CYP, children and young people.

**Table 4:** Quotations to illustrate identified themes

Themes and example concerns	Illustrative quotes*
**Labelling and dentity**	
Stigma	‘In assembly at school when there’s lots of people there. I’m taking it out, and most people have normal inhalers, and I’m pulling this massive thing out. Even the teachers would be looking at me like “what’s that?” There’d be a lot of questions especially the teachers, because they would want to know what it is and everything’. (Adolescent, exact age unknown)^25^
Cyberbullying	‘The problem with an online chatroom is you’re going to get people who don’t actually need help and they don’t need to be on the website at all. They’re like ”Hey guys, you know what would be funny, making fun of these depressed kids”’. (14 years)^14^
Inclusivity	‘With some of the apps or even like a blog and stuff, you could have a specific, um, part or like theme for disabled so that people who are like…you'd be talking to people who understand what you're going through in a way’. (12 years)^20^ ‘I personally don’t like being grouped in specifically with people with disabilities, because it makes me think I’m not normal if I’m being stuck with other people who have disabilities too. It makes me focus on the fact that I’m different, and I don’t really like that’. (Adolescent, age not stated)^16^
**Accessibility**	
Usability	‘I’ve had a look on the NHS site… it’s quite wordy and that sort of thing I wouldn’t necessarily understand… it’s sort of doctorised… it’s not necessarily aimed at young people’. (Adolescent, exact age unknown)^23^
Financial cost	‘… [Y]ou have to like buy them but that’s annoying cos they should be free…I haven’t even got a credit card’. (Adolescent, exact age unknown)^28^
Access to WiFi	‘Sometimes, when I don’t have WiFi it is hard for me’. (Exact age unknown)^45^
School rules	‘Having it [the app] in class would be helpful, cause they say you’re not allowed to have a phone in class. I can’t have it out in any of my classes … in the middle of the day, if you have trouble breathing you might want to record it so you can tell your pulmonologist’. (Age unknown)^45^
**Privacy**	
Data sharing and confidentiality	‘I don’t really like the idea of it being on Facebook… I mean people can hack into you to see what you’ve been writing and people can, without hacking into you; see what you’ve written…’ (Age unknown)^26^
Being monitored or watched	‘Hmm err it was a little bit spyee … because they are checking up to see if I’m taking my inhaler by watching me instead of asking me’. (Adolescent, exact age unknown)^30^
Control	‘I want to be very certain of exactly what they can see’. (Age not stated)^42^
**Trustworthiness and reliability**	Most of the sites regarding stuff like diet are like forums, so anyone can post, so there’s not really that much reliability…t he Kidney Foundation or something, that’s pretty reliable obviously ’cause it’s a government website, so I use that mostly’. (17 years)^27^
Discomfort or unease	‘I might not get the same level of attention and you know, kind of therapeutic qualities that I would if I was in a room with a therapist, and it’s not like personal, you know, you know what I mean, because you’re not right there with them, talking about it, you’re on a keyboard talking about it’. (Adolescent, exact age unknown)^47^
Responsiveness	
Fear of misinterpretation	‘Yeah, I mean, there’s inside jokes between me and my friends, and if he or she didn’t know about it, she [provider] might take that the wrong way… I don’t know how they [providers] would put it – as unsafe, or between me and my friends as a joke. And I wouldn’t know how they would take it’. (Age 14–17 years)^47^

*Age and terminology (eg, adolescent and child) as reported by primary study.

#### Labelling and identity

CYP were concerned that stigma could arise from technology visibility, for example, the potential for social embarrassment prevented them from using devices in public.^14^ Many technologies were designed to enable CYP to engage with an online community of users, which in some cases included other CYP from the healthy population, which led to CYPs’ concerns about cyberbullying.^14 50^ Some CYP felt that technologies involving online communities should have separate condition-specific spaces to reduce the risk of discrimination and support inclusivity.^20 44 48^ Suggestions included private messaging or chat options.^20^ Conversely, some CYP expressed concerns about technologies that exclusively brought together CYP with the same condition in forums or chatrooms.^16^

Overall, there was a tension between the need for normalisation and the risk of discrimination. For some CYP, ‘being normal’ meant feeling part of a community of other CYP who shared their condition/s and experience/s; while for others, it was also about feeling included in a community of healthy peers.

#### Accessibility

This included usability concerns regarding the age and developmental appropriateness of content^26 31 34 38^ and risks associated with bringing CYP from a broad age range together in forums or chat rooms,^28^ such as an increase in perceived ‘noise’ that might prevent individual voices being heard and understood.^16^ CYP also expressed preferences for plain language and the absence of jargon or medical terminology that they would find difficult to understand.^23 27 36^

CYP identified limited access to Wi-Fi in hospitals, at home and in the community as possible barriers to some technologies.^26 29^ Rules imposed in schools regarding mobile phone use were also highlighted.^41 45^

CYP highlighted financial costs^28 31^ associated with using mobile data^45^ to access apps as well as the impact on device storage capacity^43^ and challenged the assumption that all CYP used social media or had access to smartphones.^49^

#### Privacy

Some privacy concerns were linked to technology visibility that may draw attention to an undisclosed condition.^18 25^ CYP highlighted the potential for unwanted attention^35 44^ and questioning that may arise from using a device.^25^ Concerns surrounding data sharing and confidentiality of personal information were also evident.^14 22 33 39 48^ CYP had preferences about whom they would share data with and were concerned about the perceived dangers and negative implications of sharing data widely.^25 31 40^ For example, the risks of being ‘hacked’^13 26^ and the importance of privacy settings^24^ in various social media platforms and apps; privacy related to content that CYP created^50^ and fears of being monitored or watched by parents and/or clinicians^17 24 30 47^; and the permanence of data on websites and apps.^13^

Ultimately, CYP desired control over their data and privacy; they sought a balance between safety, confidentiality, anonymity and the option to foster connection with others by ‘putting a face to the name’^21^ and sharing personal information if they so choose.

#### Trustworthiness and reliability

CYP were generally wary of online information (through websites or apps)^27 37^ unless it was perceived to be from a trusted ‘official source’, for example, from recognisable organisations or endorsed by clinicians with expertise in their condition.^23 26 29 36 38^ They also raised concerns about images or content that could be perceived as overly negative or alarmist about their condition,^36 48^ although some CYP were concerned about images that they perceived to be unrealistic or idealised (particularly in relation to body image).^37^

Some CYP expressed discomfort or unease with the introduction of technologies that reduce face-to-face contact with their clinician. CYP were particularly concerned about the potential for lack of clinician responsiveness^19 47^ and the impact on their ability to form an open, honest and therapeutic relationship^17^ as well as the risk of clinicians missing important non-verbal cues.^13 30^

Linked to this, a general fear of misinterpretation was also identified.^47^ CYP expressed concerns that information recorded on devices (rather than in conversation) could land them in trouble with limited opportunity to explain their side of the story^30^

### Stakeholder consultation

When discussing the findings with CYP and parents, they expressed surprise at the level of concern for cyberbullying in relation to using health technologies to manage an LTC. However, they concurred with concerns identified in the review relating to security of data and information. They were surprised by studies reporting that language was not age appropriate, as they presumed that mobile apps would at least be ‘word-friendly’ for children if that was the target end user. The group noted that CYP will have different reasons and motivations for using technology and felt it was important to ensure that CYP were involved early in technology development and to not underestimate the input and impact that CYP can have. They also suggested gamification to help young children with technology. The group felt incorporating passcodes, or other forms of security, was important to ensure data security and access.

## Discussion

### Main findings

This review has highlighted CYP’s specific concerns about the use of technology to self-manage LTCs including labelling and identity; accessibility; privacy; and trustworthiness of information. Most studies were undertaken in high-income countries and mainly sought the views of CYP aged 11 years and older in relation to a wide range of health technologies. The focus on older CYP possibly reflects difficulties that researchers expect to encounter when undertaking research with children^52^ and indicates a gap in knowledge about the concerns of CYP under 11 years. The most common LTCs studied included type 1 diabetes, asthma and mental health conditions. Included studies generally had small samples. Many studies were excluded because they focused on the views and concerns of parents and/or clinicians only.

### Our findings in relation to the literature

The use of health technologies by CYP to manage LTCs is increasing with many studies describing their development, acceptability and use by CYP^45 53–61^; effectiveness^53 62–64^; and compliance by CYP.^41 57^ However, there is limited literature on the concerns that CYP may have when (or before) using a health technology for self-managing their LTC, and no review has specifically explored these concerns.

Our results indicate that the views of CYP with LTC are under-represented in the literature. Many potentially eligible studies reported solely on clinicians’ or parents’ views or failed to separate out concerns expressed by CYP and adults. As previously reported, primary studies exploring CYP’s concerns tend to involve healthy populations^63 65 66^ (eg, schoolchildren) rather than CYP with LTCs, even when evaluating the use of technologies that are designed for use by CYP with LTCs. Authentic user involvement in technology design and research is important and increasingly required by funders; CYP with LTCs are uniquely placed to explain their concerns about new technologies.

We did not find any studies examining CYP concerns regarding the use of virtual or augmented reality technologies to self-manage LTCs. This may reflect wider-reaching tendencies by researchers to only seek proxy views about how CYP use technology to manage an LTC.

Our findings are consistent with a previous review on the use of digital clinical communication (eg, telehealth) for CYP with long-term mental health conditions reporting that most studies focused only on satisfaction, acceptance or feasibility of the technology.^67^ While these issues are important, a broader focus on general concerns contributes to our understanding of potential barriers to technology use.

We identified a range of concerns, several clustered around a theme of labelling and identity and highlighting that CYP with LTCs are a diverse group, and those with the same condition may have differing concerns about the use of interactive technologies. CYP varied in whether they wanted their condition to be known, to interact with others with the same condition, or with healthy CYP. These concerns are supported by previous literature that highlights variations in how CYP wish to use online forums.^68 69^ The potential risk of cyberbullying identified in some studies is supported by a recent review about risks associated with the use of social media by CYP.^70^ In addition, CYP were particularly cautious about stigma arising from the use of technologies to manage mental health conditions and sexually transmitted infections.^71^

Accessibility of the technology, through age-appropriate language, style and physical access, was important. This concern is supported by other literature involving CYP without LTCs, for instance the ability of school-aged CYP to identify and access information about sexual health.^72^ The importance of language was also recognised as important in some studies.^69^

A not unexpected key theme in this review was privacy.^72–75^ Our findings complement a recent review calling for research that explores CYP’s privacy and data security issues when using digital health technology to manage LTCs.^76^

Trust in the technology was another important factor to determine whether CYP would use a particular technology to manage a LTC. A recent review highlighted the importance of clinicians understanding CYP’s needs in relation to their use of health technologies and also to help CYP identify appropriate technology.^4^ A study examining the concerns of CYP (without LTCs) also highlighted the concern, consistent across all age groups, of trust for health-related social media.^77^

Based on the concerns raised in the included studies within this review, we have developed a set of recommendations in conjunction with our CYP and parent stakeholders that we feel are important for future development and use of technology by CYP with LTCs (see box 1).

Box 1 **Recommendations**

The following recommendations derive from our findings and are supported by the project stakeholders:

- Ensure any technology for use by CYP is *age and developmentally appropriate* (in terms of language and style; if the technology is social media, then carefully consider the appropriate age range of participants).
- CYP will want to use technology for *different reasons* and with *different motivations* (eg, some will want to use technology that connects them to others with the same condition for support, while others will not want to be segregated by their condition). Give CYP the option of how they use technology. Technology developers should involve CYP in the design and development of health technologies.
- CYP may have concerns about using technology to manage an LTC, and *these concerns should be considered* alongside any potential benefits for CYP.
- *Trust* will be an important factor for CYP using technology for their health; they will want to know *how the technology has been developed, curated, tested and used* previously in order to make an informed decision about whether they want to use it.
- For technology involving images, recognise that CYP *may not filter what they see* and some may be surprised or concerned by distressing images (eg, on closed Facebook groups). A careful and sensitive approach should be taken to minimise CYP’s concerns.
- Consider making the technology (eg, forums or text on websites) *not overly negative*, particularly consider moderation for peer communication, to avoid causing unnecessary anxiety for end users.
- For any technology involving data, explain to CYP *who will have access to their information, how their information will be stored* and how CYP can change such access. Consider having a *passcode or biometric protection* for access to mobile apps, or where the operating system allows, prompting the use of these functions. Where messaging occurs, consider end-to-end encryption and self-destructing messages.
- Recognise that CYP are taught *digital safety* in school, including caution around sharing their information, and may feel that doing so for the purposes of health technology contradicts this. They will want to know *who will have access to their information and why*.

In addition, stakeholders recommended the following:

- *Do not under-estimate CYP’s capabilities* and the important input they can provide to technology development.
- Consider *gamification* within technologies for younger CYP with LTCs.
- When developing technology for use by CYP to manage LTCs, *involve the appropriate group of CYP early in the process* to ensure that the technology will be something they will want to use and will meet their needs. For example, if you plan to develop technology for CYP aged Y years with condition X, then work with CYP that are of this age with this condition.
- Consider whether *health inequalities may be created or exacerbated* if the technology has a financial cost associated with it.
- Tell CYP what the actual *impact of using the technology* will be for them (eg, will it help them, are there any risks).

### Strengths and limitations of the review

A strength of this review is its broad focus on technologies and LTCs in order to identify all information about CYPs’ concerns regarding use of technology to manage LTCs. We used recognised processes to ensure methodological rigour and consulted with CYP and parents. Due to the volume of records identified, we only reviewed full texts of articles that mentioned or alluded to concerns within the abstract. We did not include positive preferences such as what CYP liked or preferred (eg, design features and interactivity elements). We note that many studies focus on positive preferences of CYP for technologies, and this may be an avenue for future research. Many of the included studies were conducted in high-income countries and findings may not generalisable to CYP in low-income countries.
