# Supplementary material for: Children and young people’s concerns and needs relating to their use of health technology to self-manage long-term conditions: a scoping review
Source: Arch Dis Child. 2020 May 22;105(11):1093–104. doi: 10.1136/archdischild-2020-319103 (PMC7588410; doi:10.1136/archdischild-2020-319103)
Supplement: Supplementary data [file archdischild-2020-319103supp001.pdf]

Appendix 1 – search strategy

Databases searched:

Medline (Ovid)

PsycINFO (Ovid)

CINAHL Plus (Ebsco)

Limits: 2008 onwards, English language only

Study designs: qualitative, qualitative reviews, surveys, questionnaires, feasibility studies

Search results

**Database**	**Number of records retrieved before deduplication**	**Number of records after deduplication**
MEDLINE **ALL Ovid**	10,290	10279
PsycINFO Ovid	6968	5093
CINAHL Ebsco	7073	2993
**Total in EndNote**	**24,331**	**18365**

Search strategies

MEDLINE(R) ALL

via Ovid http://ovidsp.ovid.com/

1946 to February 8th 2019

Searched on: 11th February 2019

Records retrieved: 10279

1 exp Child/ (1809524)

2 Adolescent/ (1911611)

3 (child$ or pediat$ or paediat$ or preschool$ or pre school$ or schoolchild$ or school age$ or schoolage$ or schoolboy$ or schoolgirl$).ti,ab. (1429725)

4 (girl or girls or boy or boys or kid or kids).ti,ab. (220552)

5 (adoles$ or preadolescen$ or puberty or prepuberty or pubescen$ or prepubescen$ or teen$ or youth$ or preteen$ or juvenil$).ti,ab. (400129)

6 (young adj (people$ or person or persons)).ti,ab. (27520)

7 or/1-6 (3441306)

8 Mobile Applications/ (3730)

9 Cell Phone/ (7646)

10 Smartphone/ (2586)

11 (mhealth or m-health or mobile health).ti,ab. (3518)

12 ((mobile$ or smartphone$ or smart-phone$ or iphone$ or i-phone$ or android or ipad$ or i-pad$ or tablet$) adj2 (app or apps or application$)).ti,ab. (6756)

13 ((portab$ or electronic$ or digital$) adj2 (app or apps or application$)).ti,ab. (3393)

14 ((Health or healthcare or medical or clinical or NHS) adj2 (app or apps)).ti,ab. (1000)

15 ((mobile$ or smartphone$ or smart-phone$) adj2 technolog$).ti,ab. (2762)

16 or/8-15 (22990)

17 7 and 16 (3808)

18 virtual reality/ (665)

19 Virtual Reality Exposure Therapy/ (450)

20 ((virtual or VR) adj3 (therap$ or treatment$ or intervention$ or health or healthcare or medic$)).ti,ab. (2476)

21 18 or 19 or 20 (3379)

22 7 and 21 (487)

23 exp Telemedicine/ (24268)

24 (ehealth or e-health or etherap$ or e-therap$).ti,ab. (4145)

25 (Telemedic$ or telehealth or telehealthcare or telecare or telemental or telemanagement or telerehabilitation or telepsychology or telepsychiatry or teletherapy or tele-medic$ or tele-health or tele-healthcare or tele-care or tele-management or tele-mental or tele-rehabilitation or tele-psychology or tele-psychiatry or tele-therapy).ti,ab. (14912)

26 23 or 24 or 25 (31961)

27 7 and 26 (3728)

28 ((digital$ or digitis$ or digitiz$) adj3 (health or service$ or deliver$ or care or healthcare or intervention$ or therap$ or treatment$)).ti,ab. (4167)

29 7 and 28 (491)

30 ((digital$ or digitis$ or digitiz$) adj3 (device$ or technolog$ or tool$)).ti,ab. (4562)

31 (electronic$ adj3 (device$ or technolog$ or tool$)).ti,ab. (12919)

32 (technolog$ adj2 device$).ti,ab. (1958)

33 30 or 31 or 32 (19266)

34 7 and 33 (1516)

35 gamification.ti,ab. (283)

36 ((game$ or gaming or videogam$) adj3 (therap$ or treatment$ or intervention$ or health or healthcare or medic$)).ti,ab. (1516)

37 exergam$.ti,ab. (439)

38 (serious adj (game$ or gaming)).ti,ab. (459)

39 (gamified adj2 (app or apps or application$)).ti,ab. (17)

40 ((game$ or gaming or videogam$) adj2 (app or apps or application$)).ti,ab. (194)

41 35 or 36 or 37 or 38 or 39 or 40 (2641)

42 7 and 41 (851)

43 Augment$ realit$.ti,ab. (1352)

44 real world space$.ti,ab. (7)

45 AR device$.ti,ab. (17)

46 43 or 44 or 45 (1361)

47 7 and 46 (58)

48 exp Wearable Electronic Devices/ (9155)

49 wearable$.ti,ab. (8026)

50 ((fitness or activit$) adj2 track$).ti,ab. (1158)

51 (smartwatch$ or smart-watch$).ti,ab. (227)

52 pedometer$.ti,ab. (2385)

53 fitbit$.ti,ab. (378)

54 48 or 49 or 50 or 51 or 52 or 53 (19985)

55 7 and 54 (3727)

56 Telemetry/ (9437)

57 Remote Sensing Technology/ (2350)

58 telemetr$.ti,ab. (8066)

59 (telemonitor$ or tele-monitor$).ti,ab. (1385)

60 ((remote$ or mobile$ or electronic$) adj2 monitor$).ti,ab. (5440)

61 (remote$ adj2 (sensing or sensor or sensors or biosensor$ or technolog$)).ti,ab. (6717)

62 exp Monitoring, Physiologic/ (162870)

63 remote$.ti,ab. (66708)

64 62 and 63 (3339)

65 ((wireless$ or wifi or wi-fi) adj3 monitor$).ti,ab. (826)

66 ((wireless$ or wifi or wi-fi) adj3 (sensing or sensor or sensors or biosensor$)).ti,ab. (2935)

67 Wireless Technology/ (2996)

68 56 or 57 or 58 or 59 or 60 or 61 or 64 or 65 or 66 or 67 (32986)

69 7 and 68 (2321)

70 Text Messaging/ (2135)

71 (text messag$ or texting or short message$ or SMS or digital$ messag$ or instant messag$).ti,ab. (8435)

72 70 or 71 (8861)

73 7 and 72 (2292)

74 Social Media/ (5446)

75 social media$.ti,ab. (7227)

76 (twitter$ or tweet or tweets or tweeting or Facebook or Instagram or YouTube).ti,ab. (5780)

77 74 or 75 or 76 (12336)

78 7 and 77 (2673)

79 Blogging/ (899)

80 (Blog or blogs or blogging or weblog$).ti,ab. (1563)

81 79 or 80 (2042)

82 7 and 81 (298)

83 Therapy, Computer-Assisted/ (6426)

84 user-computer interface/ (35277)

85 (computer$ adj3 (therap$ or intervention$ or treatment$)).ti,ab. (3712)

86 83 or 84 or 85 (43944)

87 7 and 86 (4062)

88 Biomedical Enhancement/ (567)

89 Medical Informatics/ (10929)

90 Medical Informatics Applications/ (2387)

91 Automation/ (16848)

92 (technolog$ adj2 (health or healthcare or medical)).ti,ab. (19194)

93 88 or 89 or 90 or 91 or 92 (48268)

94 7 and 93 (3207)

95 17 or 22 or 27 or 29 or 34 or 42 or 47 or 55 or 69 or 73 or 78 or 82 or 87 or 94 (25721)

96 exp qualitative research/ (44025)

97 Interview/ (28345)

98 Focus Groups/ (26084)

99 Qualitative.mp. (212724)

100 Interview$.mp. (352028)

101 Experience$.mp. (970820)

102 Focus group$.ti,ab. (39514)

103 (accept$ or attitude$ or barrier$ or belief$ or believ$ or concern$ or experienc$ or facilitator$ or opinion$ or perceiv$ or perception$ or preference$ or view or views or viewpoint$).ti,ab. (2995696)

104 Feasibility Studies/ (60978)

Blower S, *et al. Arch Dis Child* 2020; 105:1093–1104. doi: 10.1136/archdischild-2020-319103

105 feasibility.ti,ab. (159011)

106 "Surveys and Questionnaires"/ (417680)

107 (survey$ or questionnaire$).ti,ab. (927022)

108 (mixed method$ or multimethod$ or multi-method$ or multi method$).mp. (19142)

109 (evidence synthes$ or realist synthes$).ti,ab. (3677)

110 (meta-synthes$ or metasynthes$).ti,ab. (989)

111 (meta-ethnograph$ or metaethnograph$).ti,ab. (463)

112 (meta-study or metastudy).ti,ab. (87)

113 realist review$.ti,ab. (253)

114 or/96-113 (4001371)

115 95 and 114 (13068)

116 exp animals/ not humans/ (4545687)

117 115 not 116 (13021)

118 limit 117 to english language (12530)

119 limit 118 to yr="2008 -Current" (10290)

PsycINFO

via Ovid http://ovidsp.ovid.com/

2002 to February Week 1 2019

Searched on: 11th February 2019

Records retrieved: 6968

1 childhood birth 12 yrs.ag. (282987)

2 preschool age 2 5 yrs.ag. (72332)

3 school age 6 12 yrs.ag. (168442)

4 adolescence 13 17 yrs.ag. (257965)

5 or/1-4 (422059)

6 pediatrics/ (21301)

7 (child$ or pediat$ or paediat$ or preschool$ or pre school$ or schoolchild$ or school age$ or schoolage$ or schoolboy$ or schoolgirl$).ti,ab. (381131)

8 (girl or girls or boy or boys or kid or kids).ti,ab. (56002)

9 (adoles$ or preadolescen$ or puberty or prepuberty or pubescen$ or prepubescen$ or teen$ or youth$ or preteen$ or juvenil$).ti,ab. (202921)

10 (young adj (people$ or person or persons)).ti,ab. (23865)

11 6 or 7 or 8 or 9 or 10 (519844)

12 5 or 11 (634745)

13 mobile devices/ (2155)

14 cellular phones/ (4208)

15 (mhealth or m-health or mobile health).ti,ab. (714)

16 ((mobile$ or smartphone$ or smart-phone$ or iphone$ or i-phone$ or android or ipad$ or i-pad$ or tablet$) adj2 (app or apps or application$)).ti,ab. (2258)

17 ((portab$ or electronic$ or digital$) adj2 (app or apps or application$)).ti,ab. (228)

18 ((Health or healthcare or medical or clinical or NHS) adj2 (app or apps)).ti,ab. (189)

19 ((mobile$ or smartphone$ or smart-phone$) adj2 technolog$).ti,ab. (1926)

20 13 or 14 or 15 or 16 or 17 or 18 or 19 (8682)

21 12 and 20 (2083)

22 virtual reality/ (6907)

23 ((virtual or VR) adj3 (therap$ or treatment$ or intervention$ or health or healthcare or medic$)).ti,ab. (1099)

24 22 or 23 (7296)

25 12 and 24 (1008)

26 telemedicine/ (4597)

27 (ehealth or e-health or etherap$ or e-therap$).ti,ab. (1225)

28 (Telemedic$ or telehealth or telehealthcare or telecare or telemental or telemanagement or telerehabilitation or telepsychology or telepsychiatry or teletherapy or tele-medic$ or tele-health or tele-healthcare or tele-care or tele-management or tele-mental or tele-rehabilitation or tele-psychology or tele-psychiatry or tele-therapy).ti,ab. (3070)

29 26 or 27 or 28 (6091)

30 12 and 29 (1001)

31 ((digital$ or digitis$ or digitiz$) adj3 (health or service$ or deliver$ or care or healthcare or intervention$ or therap$ or treatment$)).ti,ab. (716)

32 12 and 31 (164)

33 exp medical therapeutic devices/ (5157)

34 (digital$ or digitis$ or digitiz$ or electronic$ or technolog$).ti,ab. (115782)

35 33 and 34 (726)

36 ((digital$ or digitis$ or digitiz$) adj3 (device$ or technolog$ or tool$)).ti,ab. (3025)

37 (electronic$ adj3 (device$ or technolog$ or tool$)).ti,ab. (1414)

38 (technolog$ adj2 device$).ti,ab. (540)

39 35 or 36 or 37 or 38 (5547)

40 12 and 39 (1476)

41 gamification.ti,ab. (334)

42 ((game$ or gaming or videogam$) adj3 (therap$ or treatment$ or intervention$ or health or healthcare or medic$)).ti,ab. (891)

43 exergam$.ti,ab. (238)

44 (serious adj (game$ or gaming)).ti,ab. (543)

45 (gamified adj2 (app or apps or application$)).ti,ab. (16)

46 ((game$ or gaming or videogam$) adj2 (app or apps or application$)).ti,ab. (210)

47 41 or 42 or 43 or 44 or 45 or 46 (2047)

48 12 and 47 (708)

49 Augment$ realit$.ti,ab. (518)

50 real world space$.ti,ab. (6)

51 AR device$.ti,ab. (7)

52 49 or 50 or 51 (524)

53 12 and 52 (112)

54 wearable$.ti,ab. (773)

55 ((fitness or activit$) adj2 track$).ti,ab. (305)

56 (smartwatch$ or smart-watch$).ti,ab. (54)

57 pedometer$.ti,ab. (753)

58 fitbit$.ti,ab. (91)

59 54 or 55 or 56 or 57 or 58 (1861)

60 12 and 59 (401)

61 telemetry/ (98)

62 telemetr$.ti,ab. (676)

63 (telemonitor$ or tele-monitor$).ti,ab. (159)

64 ((remote$ or mobile$ or electronic$) adj2 monitor$).ti,ab. (839)

65 (remote$ adj2 (sensing or sensor or sensors or biosensor$ or technolog$)).ti,ab. (278)

66 exp monitoring/ (11444)

67 remote$.ti,ab. (8129)

68 66 and 67 (139)

69 ((wireless$ or wifi or wi-fi) adj3 monitor$).ti,ab. (46)

70 ((wireless$ or wifi or wi-fi) adj3 (sensing or sensor or sensors or biosensor$)).ti,ab. (163)

71 61 or 62 or 63 or 64 or 65 or 68 or 69 or 70 (2156)

72 12 and 71 (282)

73 text messaging/ (723)

74 (text messag$ or texting or short message$ or SMS or digital$ messag$ or instant messag$).ti,ab. (3820)

75 73 or 74 (3917)

76 12 and 75 (1222)

77 exp social media/ (11686)

78 social media$.ti,ab. (8331)

79 (twitter$ or tweet or tweets or Facebook).ti,ab. (5895)

80 77 or 78 or 79 (16221)

81 12 and 80 (3307)

82 blog/ (416)

83 (Blog or blogs or blogging or weblog$).ti,ab. (2921)

84 82 or 83 (2986)

85 12 and 84 (523)

86 computer assisted therapy/ (978)

87 (computer$ adj3 (therap$ or intervention$ or treatment$)).ti,ab. (1642)

88 86 or 87 (2324)

89 12 and 88 (605)

90 computer applications/ (4570)

91 automation/ (1375)

92 (technolog$ adj2 (health or healthcare or medical)).ti,ab. (2755)

93 90 or 91 or 92 (8631)

94 12 and 93 (1068)

95 21 or 25 or 30 or 32 or 40 or 48 or 53 or 60 or 72 or 76 or 81 or 85 or 89 or 94 (11934)

96 qualitative research/ (7731)

97 interviews/ (4954)

98 interviewing/ (1632)

99 experience$.mp. (438785)

100 interview$.mp. (287976)

101 qualitative$.mp,md. (256517)

102 Focus group$.ti,ab. (29135)

103 (accept$ or attitude$ or barrier$ or belief$ or believ$ or concern$ or experienc$ or facilitator$ or opinion$ or perceiv$ or perception$ or preference$ or view or views or viewpoint$).ti,ab. (1062112)

104 feasibility.ti,ab. (16513)

105 exp surveys/ (5509)

106 exp questionnaires/ (10863)

107 (survey$ or questionnaire$).ti,ab. (342254)

108 (mixed method$ or multimethod$ or multi-method$ or multi method$).mp. (24452)

109 (evidence synthes$ or realist synthes$).ti,ab. (611)

110 (meta-synthes$ or metasynthes$).ti,ab,md. (784)

111 (meta-ethnograph$ or metaethnograph$).ti,ab. (266)

112 (meta-study or metastudy).ti,ab. (72)

113 realist review$.ti,ab. (56)

114 96 or 97 or 98 or 99 or 100 or 101 or 102 or 103 or 104 or 105 or 106 or 107 or 108 or 109 or 110 or 111 or 112 or 113 (1386188)

115 95 and 114 (8010)

116 limit 115 to (english language and yr="2008 -Current") (6968)

Cumulative Index to Nursing & Allied Health (CINAHL Plus)

via EBSCO https://www.ebscohost.com/

Inception to 8th February 2019

Searched on: 11th February 2019

Records retrieved: 7073

S1	(MH "Child+")	566,309
S2	(MH "Adolescence+")	447,771
S3	TI (child* or pediat* or paediat* or preschool* or pre school* or schoolchild* or school age* or schoolage* or schoolboy* or schoolgirl*) OR AB (child* or pediat* or paediat* or preschool* or pre school* or schoolchild* or school age* or schoolage* or schoolboy* or schoolgirl*)	456,968
S4	TI (girl or girls or boy or boys or kid or kids) OR AB (girl or girls or boy or boys or kid or kids)	54,870
S5	TI (adoles* or preadolescen* or puberty or prepuberty or pubescen* or prepubescen* or teen* or youth* or preteen* or juvenil*) OR AB (adoles* or preadolescen* or puberty or prepuberty or pubescen* or prepubescen* or teen* or youth* or preteen* or juvenil*)	154,735
S6	TI (young N1 (people* or person or persons)) OR AB (young N1 (people* or person or persons))	16,397
S7	S1 OR S2 OR S3 OR S4 OR S5 OR S6	995,486
S8	(MH "Mobile Applications")	4,780
S9	(MH "Cellular Phone")	1,489
S10	(MH "Smartphone")	1,821
S11	TI (mhealth or m-health or "mobile health") OR AB (mhealth or m-health or "mobile health")	1,886
S12	TI (((mobile* or smartphone* or smart-phone* or iphone* or i-phone* or android or ipad* or i-pad* or tablet*) N2 (app or apps or application*))) OR AB (	4,457

Blower S, *et al. Arch Dis Child* 2020; 105:1093–1104. doi: 10.1136/archdischild-2020-319103

	((mobile* or smartphone* or smart-phone* or iphone* or i-phone* or android or ipad* or i-pad* or tablet*) N2 (app or apps or application*)))	
S13	TI (((portab* or electronic* or digital*) N2 (app or apps or application*))) OR AB (((portab* or electronic* or digital*) N2 (app or apps or application*)))	559
S14	TI (((health or healthcare or medical or clinical or NHS) N2 (app or apps))) OR AB (((health or healthcare or medical or clinical or NHS) N2 (app or apps)))	861
S15	TI (((mobile* or smartphone* or smart-phone*) N2 technolog*)) OR AB (((mobile* or smartphone* or smart-phone*) N2 technolog*))	1,819
S16	S8 OR S9 OR S10 OR S11 OR S12 OR S13 OR S14 OR S15	12,343
S17	S7 AND S16	1,238
S18	(MH "Virtual Reality")	3,703
S19	(MH "Virtual Reality Exposure Therapy")	39
S20	TI (((virtual or VR) N3 (therap* or treatment* or intervention* or health or healthcare or medic*))) OR AB (((virtual or VR) N3 (therap* or treatment* or intervention* or health or healthcare or medic*)))	1,547
S21	S18 OR S19 OR S20	4,842
S22	S7 AND S21	651
S23	(MH "Telehealth+")	18,252
S24	TI (ehealth or e-health or etherap* or e-therap*) OR AB (ehealth or e-health or etherap* or e-therap*)	2,811
S25	TI (Telemedic* or telehealth or telehealthcare or telecare or telemental or telemanagement or telerehabilitation or telepsychology or telepsychiatry or teletherapy or tele-medic* or tele-health or tele-healthcare or tele-care or tele-management or tele-mental or tele-rehabilitation or tele-psychology or tele-psychiatry or tele-therapy) OR AB (Telemedic* or telehealth or telehealthcare or telecare or telemental or telemanagement or telerehabilitation or telepsychology or telepsychiatry or teletherapy or tele-medic* or tele-health or tele-healthcare or tele-care or tele-management or tele-mental or tele-rehabilitation or tele-psychology or tele-psychiatry or tele-therapy)	7,901
S26	S23 OR S24 OR S25	22,015
S27	S7 AND S26	2,484
S28	TI (((digital* or digitis* or digitiz*) N3 (health or service* or deliver* or care or healthcare or intervention* or therap* or treatment*))) OR AB (((digital* or	2,153

	digitis* or digitiz*) N3 (health or service* or deliver* or care or healthcare or intervention* or therap* or treatment*)))	
S29	S7 AND S28	258
S30	TI (((digital* or digitis* or digitiz*) N3 (device* or technolog* or tool*))) OR AB (((digital* or digitis* or digitiz*) N3 (device* or technolog* or tool*)))	2,161
S31	TI ((electronic* N3 (device* or technolog* or tool*))) OR AB ((electronic* N3 (device* or technolog* or tool*)))	3,247
S32	TI (technolog* N2 device*) OR AB (technolog* N2 device*)	960
S33	S30 OR S31 OR S32	6,280
S34	S7 AND S33	944
S35	TI gamification OR AB gamification	221
S36	TI (((game* or gaming or videogam*) N3 (therap* or treatment* or intervention* or health or healthcare or medic*))) OR AB (((game* or gaming or videogam*) N3 (therap* or treatment* or intervention* or health or healthcare or medic*)))	991
S37	TI exergam* OR AB exergam*	232
S38	TI ((serious N1 (game* or gaming))) OR AB ((serious N1 (game* or gaming)))	263
S39	TI ((gamified N2 (app or apps or application*))) OR AB ((gamified N2 (app or apps or application*)))	22
S40	TI (((game* or gaming or videogam*) N2 (app or apps or application*))) OR AB (((game* or gaming or videogam*) N2 (app or apps or application*)))	140
S41	S35 OR S36 OR S37 OR S38 OR S39 OR S40	1,716
S42	S7 AND S41	530
S43	TI Augment* N1 realit* OR AB Augment* N1 realit*	456
S44	TI real world space* OR AB real world space*	11
S45	TI ("AR device" or "AR devices") OR AB ("AR device" or "AR devices")	5
S46	S43 OR S44 OR S45	467
S47	S7 AND S46	41
S48	(MH "Wearable Sensors+")	3,838

Blower S, *et al. Arch Dis Child* 2020; 105:1093–1104. doi: 10.1136/archdischild-2020-319103

S49	TI wearable* OR AB wearable*	1,873
S50	TI (((fitness or activit*) N2 track*)) OR AB (((fitness or activit*) N2 track*))	591
S51	TI (smartwatch* or smart-watch*) OR AB (smartwatch* or smart-watch*)	112
S52	TI pedometer* OR AB pedometer*	1,394
S53	TI fitbit* OR AB fitbit*	237
S54	S48 OR S49 OR S50 OR S51 OR S52 OR S53	6,671
S55	S7 AND S54	1,434
S56	(MH "Telemetry")	1,768
S57	(MH "Biosensors")	271
S58	TI telemetr* OR AB telemetr*	1,448
S59	TI ((telemonitor* or tele-monitor*)) OR AB ((telemonitor* or tele-monitor*))	681
S60	TI (((remote* or mobile* or electronic*) N2 monitor*)) OR AB (((remote* or mobile* or electronic*) N2 monitor*))	2,311
S61	TI ((remote* N2 (sensing or sensor or sensors or biosensor* or technolog*))) OR AB ((remote* N2 (sensing or sensor or sensors or biosensor* or technolog*)))	365
S62	(MH "Monitoring, Physiologic+")	86,894
S63	TI remote* OR AB remote*	12,176
S64	S62 AND S63	864
S65	TI (((wireless* or wifi or wi-fi) N3 monitor*)) OR AB (((wireless* or wifi or wi-fi) N3 monitor*))	262
S66	TI (((wireless* or wifi or wi-fi) N3 (sensing or sensor or sensors or biosensor*))) OR AB (((wireless* or wifi or wi-fi) N3 (sensing or sensor or sensors or biosensor*)))	261
S67	(MH "Wireless Communications")	10,236
S68	(S56 OR S57 OR S58 OR S59 OR S60 OR S61 OR S64 OR S65 OR S66 OR S67)	16,551
S69	S7 AND S68	1,869
S70	(MH "Text Messaging")	1,986

S71	(MH "Instant Messaging")	189
S72	TI ((text N1 messag* or texting or short N1 message* or SMS or digital* N1 messag* or instant N1 messag*)) OR AB ((text N1 messag* or texting or short N1 message* or SMS or digital* N1 messag* or instant N1 messag*))	3,574
S73	S70 OR S71 OR S72	4,472
S74	S7 AND S73	1,335
S75	(MH "Social Media")	10,206
S76	TI social N1 media* OR AB social N1 media*	7,706
S77	TI (twitter* or tweet or tweets or tweeting or Facebook or Instagram or YouTube) OR AB (twitter* or tweet or tweets or tweeting or Facebook or Instagram or YouTube)	6,105
S78	S75 OR S76 OR S77	17,001
S79	S7 AND S78	2,796
S80	(MH "Blogs")	2,820
S81	TI (Blog or blogs or blogging or weblog*) OR AB (Blog or blogs or blogging or weblog*)	2,110
S82	S80 OR S81	4,032
S83	S7 AND S82	300
S84	(MH "Therapy, Computer Assisted")	4,961
S85	(MH "User-Computer Interface")	9,081
S86	TI ((computer* N3 (therap* or intervention* or treatment*))) OR ((computer* N3 (therap* or intervention* or treatment*)))	6,929
S87	S84 OR S85 OR S86	15,646
S88	S7 AND S87	2,117
S89	(MH "Biomedical Enhancement")	38
S90	(MH "Medical Informatics")	3,880
S91	(MH "Health Informatics")	3,230

Blower S, *et al. Arch Dis Child* 2020; 105:1093–1104. doi: 10.1136/archdischild-2020-319103

S92	(MH "Automation")	5,033
S93	TI ((technolog* N2 (health or healthcare or medical))) OR AB ((technolog* N2 (health or healthcare or medical)))	11,637
S94	S89 OR S90 OR S91 OR S92 OR S93	22,849
S95	S7 AND S94	1,593
S96	S17 OR S22 OR S27 OR S29 OR S34 OR S42 OR S47 OR S55 OR S69 OR S74 OR S79 OR S83 OR S88 OR S95	15,324
S97	(MH "Qualitative Studies")	93,665
S98	(MH "Interviews+")	192,311
S99	(MH "Focus Groups")	37,283
S100	TI qualitative OR AB qualitative	97,263
S101	TI interview* OR AB interview*	168,461
S102	TI experience* OR AB experience*	323,915
S103	TI Focus N1 group* OR AB Focus N1 group*	27,632
S104	TI ((accept* or attitude* or barrier* or belief* or believ* or concern* or experienc* or facilitat* or opinion* or perceiv* or percept* or preference* or view or views or viewpoint*)) OR AB ((accept* or attitude* or barrier* or belief* or believ* or concern* or experienc* or facilitat* or opinion* or perceiv* or percept* or preference* or view or views or viewpoint*))	871,898
S105	(MH "Pilot Studies")	66,935
S106	TI feasibility OR AB feasibility	36,553
S107	(MH "Surveys") OR (MH "Survey Research")	143,259
S108	(MH "Questionnaires+")	345,320
S109	TI ((survey* or questionnaire*)) OR AB ((survey* or questionnaire*))	347,156
S110	(MH "Multimethod Studies")	11,788
S111	TI ((mixed N1 method* or multimethod* or multi-method*)) OR AB ((mixed N1 method* or multimethod* or multi-method*))	13,905
S112	(MH "Meta Synthesis")	1,326

S113	TI (evidence N1 synthes* or realist N1 synthes*) OR AB (evidence N1 synthes* or realist N1 synthes*)	2,437
S114	TI (meta-synthes* or metasynthes*) OR AB (meta-synthes* or metasynthes*)	875
S115	TI (meta-ethnograph* or metaethnograph*) OR AB (meta-ethnograph* or metaethnograph*)	355
S116	TI (meta-study or metastudy) OR AB (meta-study or metastudy)	69
S117	TI realist N1 review* OR AB realist N1 review*	176
S118	S97 OR S98 OR S99 OR S100 OR S101 OR S102 OR S103 OR S104 OR S105 OR S106 OR S107 OR S108 OR S109 OR S110 OR S111 OR S112 OR S113 OR S114 OR S115 OR S116 OR S117	1,370,237
S119	S96 AND S118	8,110
S120	S96 AND S118<br>Limiters - Published Date: 20080101-20191231; English Language	7,073
