# Supplementary material for: Children and young people’s concerns and needs relating to their use of health technology to self-manage long-term conditions: a scoping review
Source: Arch Dis Child. 2020 May 22;105(11):1093–104. doi: 10.1136/archdischild-2020-319103 (PMC7588410; doi:10.1136/archdischild-2020-319103)
Supplement: Supplementary data [file archdischild-2020-319103supp002.pdf]

Appendix 2

Lead author and date	Study participants' age (years): range	Study participants: Long-term health condition	Type of technology	Quotes that support the concerns listed (if applicable)
Barnfather (2011)	12-18	Cerebral palsy and spina bifida	Online support	I personally don't like being grouped in specifically with people with disabilities, because it makes me think I'm not normal if I'm being stuck with other people who have disabilities too. It makes me focus on the fact that I'm different, and I don't really like that.<br><br>Well a lot of them were really young. They were like 14. {participant SB-69, 16 years}<br><br>Being 16, I'm into...going out and doing stuff, whereas the online support group kids tended to be more centred around - I don't even know how to put it- like wrestling, and all that stuff. Because I go to high school, I'm experiencing a lot deeper things than that [participant 17]
Bevan Jones (2018)	13-18	Depression	Web-based psychoeducation multimedia programme (MoodHwb)	I don’t think it should be on social media—it can be so toxic. [Young person focus group 2]

Appendix 2

Boydell (2010)	7-18 (7-18)	ADHD, Mood disorder, learning disability, anxiety disorder, attachment disorder, oppositional defiant disorder, developmental disability, fetal alcohol disorder, personality disorder, psychotic disorder, adjustment disorder (2 also had sub abuse and dependence, 13 had psychosocial issues and 6 had problems of abuse and neglect)	Telepsychiatry	I didn't like the fact that my parents were there. I don't know, there were some things that I would have preferred discussing without my parents' presence. (F, 13 year old)<br><br>You don't really know the person. You can't really judge by that…how can you help someone that you don't really know? (F, 14 year old)<br><br>Well, I think it could be useful but it would have to be more repetitive because just an hour, it's not enough. It didn't help me. (F, 18)
Bradford (2015)	12-18 (12-25)	Mental health	Electronic mental health assessment	If you had someone in front of you, you're able to understand a lot more about them just through their body language and what-not. So like if you were to be writing something down, I reckon someone's more likely to lie through that. (Female, 15–18 years)

2

Appendix 2

Brigden (2018)	12-17 (12-17)	Chronic fatigue syndrome and myalgic encephalomyelitis (CFS/ME)	Online resources	P1: the NHS are the ones that you can trust more… you know that they're like safe to be reading, like with the information you know it's probably going to be true.<br><br>P4: I've had a look on the NHS site…it's quite wordy and that sort of thing I wouldn't necessarily understand…it's sort of doctorised…it's not necessarily aimed at young people.
Britto (2012)	13-18 (13-18)	Asthma	Text messaging on mobile phone	n/a
Cafazzo (2012)	12-16 (12-16)	Type 1 diabetes	mHealth app for self-management of type 1 diabetes	None
Cai (2017)	10-18 (10-23)	Juvenile idiopathic arthritis	A smartphone app (JIApp) to improve self-management of Juvenile Idiopathic Arthritis	Arthritis is quite personal so some things you might not want to share with other people. [Patient 6; 10 years]
Carpenter (2016)	12-16 (12-16)	Asthma	Apps for self-management of asthma	None

3

Appendix 2

Clark (2018)	12-18 (12-18)	Anxiety (with or without depression)	Online mental health treatment programme	The problem with an online chatroom is you're going to get people who don't actually need help and they don't need to be on the website at all. They're like "Hey guys, you know what would be funny, making fun of these depressed kids (focus group member, aged 14).<br><br>It's just what guys do sometimes [make fun of you for seeking help] (clinical participant, aged 14, sought help from a child and adolescent mental health clinic).<br><br>"just like do it on your own terms" (clinical participant, aged 14).
Dominguez (2017)	14-18	cancer	Internet and social networks for information about their condition	R20: I never really searched, not really. I've been curious but… what if it comes back? I prefer not knowing anything or asking people to seeing whatever that could even be a lie. I don't trust Internet information (17 years old).
Donzelli (2017)	NR	idiopathic scoliosis	Thermobrace plus sensor with reading software	n/a
Dulli (2018)	15-18 (15-19)	HIV	Online support group for HIV	At first, I was very scared. I was like what if someone should just carry my phone and see and say ha! what is this? But I was scared,…later on I became used to it, I wasn't afraid if someone should pick up my phone and see it. [high engager, 18-year-old female]<br><br>There's something I didn't like because we were 8 in number in that group and anytime or sometimes when I go online, I will only see only one chat or sometimes I won't see anybody. [high engager, 16-year-old male]

Appendix 2

Holmberg (2018)	13-16	Obesity	Online weight, food and health information	"one has heard a lot … that this diet works … and then after a month it does not work … one is easily fooled online" (16/F), and "one site says this, and the other site says something else … therefore I chose not to look … it just makes me confused" (15/F)<br><br>When using Google: "I read the text, the green one …<br>the link … to see if it links to a professional site" (14/F)a<br><br>"so, it seems trustworthy … they include like interviews with others with this condition … and I know how it is to have this condition, and it matches with what I read on these blogs" (15/F)<br><br>On female fitness bloggers: "well, I feel, I get jealous sort of… that's how I feel … I am happy for their sake, but still, I can feel like no, it will never work and stuff like that … and that is not so nice" (14/F)<br><br>"it is like advertisements sort of … and if it is shown often then it gets stuck … and then you start to drink these [points at an Instagram depicting energy drinks] and that is not good for me" (15/M)

Appendix 2

Howard (2017)	11-16	Asthma	Electronic monitoring device for asthma	“I’d share it with my doctors and nurses, parents and guardians, maybe cousins and maybe if they got used to it, some really close friends who I know won’t laugh at it … I’ve got a couple of friends who I often fall out with, so I probably wouldn’t like to share it with them. And strangers, because stranger danger. People who’d laugh at it.” (Ad7)<br>"they'll probably take the mick. They'll think I have really bad disabilities, they'll think I've just got problems… if people had a problem with me, maybe the thing they'd get me with is that."<br>"At primary school when I had the spacer for my inhaler, everyone used to stare at me. So it would probably be the same thing"<br>"Because sometimes I need to carry it outside with me, because sometimes I get really wheezy during PE so I need to take it. And when I take it out people always stare at me. So they'll stare at me even more with that"<br>"I just wouldn't take it around with them (friends)... they'd ask loads of questions... you can tell it's different- not meant to be on there. Everyone would be like 'what's that? what's that?'"<br>"In assembly at school when there's lots of people there. I'm taking it out, and most people have normal inhalers, and I'm pulling this massive thing out. Even the teachers would be looking at me like 'what's that?' There'd be a lot of questions especially the teachers, becasue they would want to know what it is and everything".<br>"If you become unpopular with your friends and they start laughing at you for having something weird clipped around your inhaler"<br>"I wouldn't really be very comfortable with it, some other people who just have asthma like me just have a normal inhaler, and the I've just got this technical one, which seems a bit awkward"<br>"I've got a couple of friends who I often fall out with, so I probably wouldn't like to share it with them. And strangers, because stranger danger. People who'd laugh at it".

Appendix 2

Huby (2017)	5-17 (5-17)	chronic kidney disease	web-based support for CKD	…Having useful information. Not just everything there, just the main stuff… (Participant/038)<br><br>No not for me, I'd rather just keep it to myself and get on with it (in relation to using social media). (Participant/142)<br><br>No, it's not really about being colourful or not, it's just about the information that's there… (Participant/038)<br><br>…they might not be the right thing, they might just be about – I don't know – someone who's done a blog or something like that. (Participant/047)<br><br>I don't really like the idea of it being on Facebook…I mean people can hack into you to see what you've been writing and people can, without hacking into you; see what you've written… (Participant/047)<br><br>…I mean at my school, only my best, best friends know that I've got a problem…But I mean, everyone else doesn't know… (Participant/047)
Jibb (2018)	12-17	Cancer	App	The part where you get the advice from the nurse was good but then sometimes I would just miss her if I was out or my phone was on silent. So, it might be better if she left you a (text) message so that you could check what she was telling you to do. [Male, 14 years]

7

Appendix 2

Knibbe (2018)	12-18	Cerebral palsy	Social technologies (e.g. FB, Youtube, pedometer, fitness app, active video games)	With some of the apps or even like a blog and stuff, you could have a specific, um, part or like theme for disabled so that people who are like, perople who don't understand, you're not like, wow that's so easy, I could do that in two seconds, but you'd be talking to people who understand what you're going through in a way (Kallie, 12).<br><br>...we need to be careful about prioritising [or segregating] this, because we don't it to be so private that it can't be social anymore, you know?...not only do we want to do exercise and show people, we want to be recognized, included, accepted. (William, 15)
Maurice-Stam (2014)	11-17	Cancer	Website with secure chat room	None
Mulvaney (2013)	12-18	Asthma	Using phone to monitor asthma	
Nicholas (2009)	NR	chronic kidney disease	Online social support network	

Appendix 2

Nightingale (2017)	5-18	chronic kidney disease	Apps, websites to support CKD	Most of the sites regarding stuff like diet are like forums, so anyone can post, so there's not really that much reliability...the Kidney Foundation or something, that's pretty reliable obviously 'cause it's a government website, so I use that mostly. [Young person, aged 17 years]<br><br>I don't really like looking at the websites...because it reminds me of how much I'm different from all the rest of my friends. [Child, aged 8 years]<br><br>I think an app would probably be better, rather than going on a website to do it, because apps are more convenient. You don't have to type anything up and you can just click on it. [Young person, aged 16 years]
Nordfeldt (2013)	10-17	Type 1 diabetes	Internet use, social networking related to diabetes	Well…if you feel certain about what kind of website it is, who's behind it, then you can trust that there's no false things there, then that's good, and maybe you don't have to call the hospital and ask questions (M) Like, if you have made an appointment through the Internet to meet somebody, it may not be the person you expected (B) It could be a psycho, it could be any person, you never know, it's quite uncertain (A) Maybe a little different to add, but no, …no one I don't know, I might, it could be a paedophile, you never know really. That's what's so scary (A). That's how it is, the worst there is if there are anonymous persons there… if you're serious yourself about something and an anonymous person comments one gets angry, the other one could write just about anything because the anonymity shelters him (F)<br><br>-But like this, there shouldn't be too much text, because then I think people will get tired (M) Well, maybe a little more children's facts so to speak, with words that are easy to understand (I)

Appendix 2

Powell (2017)	8-13	ADHD	Mobile apps designed for CYP with ADHD	...you have to like buy them but that's annoying cos they should be free...I haven't even got a credit card. [YP2]<br><br>A bit childish, does it say for 13 [years old]? [YP2]<br><br>...how I didn't do anything! This app is fake! Its fake! [the app said he had achieved the next level and he hadn't done anything]. [YP3]<br><br>I didn't like it seriously, it's mind controlling! [as above, the app said she had achieved the next level and he hadn't done anything]. [YP5]
Ramsey (2018)	13-18	Asthma	Health technology for asthma management (apps mainly)	"Only asthma doctors…should see (data)"<br>"Only want the doctor to see my information in preparation for my appointment"<br><br>"I want to be very certain of exactly what they can see"
Raval (2017)	? NR (3-16)	Colorectal diseases	Apps	None
Rivera (2018)	12-18	Obesity	Mobile app for managing weight and health	Just some personal experience with apps like this. I find that you start off really well with tracking, but overtime you forget or you don't put in accurate information. So it is really easy when it comes to tracking to lose focus. (Lily, adolescent)<br><br>There could be an option to make it anonymous, or a direct message, or if you want you could put it to the group chat. (Heather, adolescent)

Appendix 2

Roberts (2016)	12-16	Asthma	Two apps for self-management of asthma	I don't really -- I don't really want my friends to know what kind of medications I'm taking, cause I like to keep it to myself. So I don't really want them knowing, cause like a lot of my friends don't even know I take so many medications anyways, like a lot of people think I'm just fine, so I'd only like trust my parents with that. (Female, 16)<br><br>Um, I mean, I think I could get my friends who have asthma to do it, but I don't know how I'd get friends without asthma to do it. You know? Cause I don't really…I don't know why they'd want to go if they don't -- on the app if they don't have asthma. (Female, age 16).
Schneider (2019)	12-17	Asthma	App for self-management of asthma	"Having it [the app] in class would be helpful, cause they say you're not allowed to have a phone in class. I can't have it out in any of my classes … in the middle of the day, if you have trouble breathing you might want to record it so you can tell your pulmonologist."<br><br>"It's hard sometimes to use it, because when you change the password, you have to enter it a couple times until it submits it. And sometimes when you can't get through, it has it locked down for like 15minutes, so then I have to wait 15minutes and then I log in." "Sometimes, when I don't have Wi-Fi it is hard for me to document."
Simons (2018)	12-13	ADHD	Remote monitoring technology	None
Stewart (2018)	11-15	Asthma	Electronic monitoring devices	Hmm err it was a little bit spyee ... . because they are checking up to see if I'm taking my inhaler by watching me instead of asking me. (Sam, adolescent)<br><br>It feels scary cause whenever I don't, whenever I think of taking it but I haven't it's like oh, whenever your found out or someone says you haven't done this and you plead innocence they are always gonna say that they won't believe you cause it's the results and you say ok I'd thought I'd taken it but I didn't know if I had and if didn't have it it was like oh they won't know so yeh I could take it then fine but then now it's like oh if I don't take it I'll be in trouble. (Gary, adolescent)

Appendix 2

Thabrew (2016)	8-17	Long-term physical conditions (CF, Chrohn's, diabetes, epilepsy, arthritis, liver disease, beta thalassaemia, bronchiesctasis, migraines, recurrent pneumothorax, non-specified 1)	eHealth interventions (online information, support and e-therapy)	None
Vaala (2018)	13-17	Type 1 diabetes	Sharing personal data with peers	n/a

Appendix 2

Van Rensburg (2016)	14-18	Variety of illnesses - ADHD, MDD, PTSD, Mood NOS, ODD, anxiety disorder, learning disability (these were all classified by the study as psychiatric illnesses)	Social media use relating to psychiatric illness	Participant 14 [17 year old female, Post-traumatic Stress Disorder (PTSD)_ and Mood Disorder NOS] I mean, what if they're not there to answer you, and you have to wait a day or so, to see if they reply. I mean, if you need an answer now, you can call them up, and talk to them, but if you Facebook message them, they won't, they probably don't even have a Facebook, you don't know.<br><br>Yeah, I mean, there's inside jokes between me and my friends, and if he or she didn't know about it, she [provider] might take that the wrong way... I don't know how they [providers] would put it – as unsafe, or between me and my friends as a joke. And I wouldn't know how they would take it. [Participant 14–17 year old female, PTSD and mood disorder NOS]<br><br>Um, I might not get the same level of attention and you know, kind of therapeutic qualities that I would if I was in a room with a therapist, and it's not like personal, you know, you know what I mean, because you're not right there with them, talking about it, you're on a keyboard talking about it, so. And a therapist can't really read you, or your texts or how you're feeling so, the way, like I could respond, and, like another hard thing is like I'm a sarcastic person, so that's, that's a hard thing to figure out when you're typing, because people don't know what you mean sometimes, it's kinda like, do you mean that literally or sarcastically? [Participant 11–14 year old female, MDD and oppositional defiant disorder (ODD)]<br><br>Um, I don't know, maybe, because like, being face to face with them, I feel like I'm more inclined to tell them the truth, whereas, I could hide my um, emotions and feelings more over social media, because like I know when I talk to my psychiatrist, she's always like, what was that face? Like what did you make that face for? And then I have to tell her, so, I think it could be problematic [Participant 18–16 year old female, MDD]<br><br>Like sometimes, I post, like on Instagram, like a picture, like of like a poem, and it's like numb, and it talks about like self-harm and stuff like that. Sometimes I post stuff like that, and if my mom or my therapist saw that, like they would freak out. [Participant 5 – 15 year old female, psychosis NOS and PTSD]

Appendix 2

Waite-Jones (2018)	10-18	Juvenile arthritis	Apps	So that people who do not have the code and do not have arthritis can't get on, so it is as easy as that. [YP1]<br><br>If somebody who did not actually have it [arthritis] but just pretended to have it to talk to children, well that could be a problem. [YP7]
Woolford (2013)	13-18	Obesity	Social media (Facebook) for weight management	[It should be secret] Not like I'm embarrassed but then no one will be all in your business.<br><br>Well I wouldn't want to have my friends see that I'm in this program—it's embarrassing. I mean I told a lot of my friends but I'm friends with almost everybody at my school so I don't know.
Wuthrich (2012)	14-17	Anxiety	Cool Teens (cCBT) - computerised program for adolescent anxiety	None
Yi-Frazier (2015)	14-18	Type 1 diabetes	Instagram for type 1 diabetes	None

Blower S, *et al. Arch Dis Child* 2020; 105:1093–1104. doi: 10.1136/archdischild-2020-319103
